# Increased Age-Dependent Risk of Death Associated With *lukF-PV*-Positive *Staphylococcus aureus* Bacteremia

**DOI:** 10.1093/ofid/ofw220

**Published:** 2016-12-05

**Authors:** Trine A. Knudsen, Robert Skov, Andreas Petersen, Anders R. Larsen, Thomas Benfield, for the Danish Staphylococcal Bacteremia Study Group

**Affiliations:** ^1^Department of Infectious Diseases and; ^2^Clinical Research Centre, Copenhagen University Hospital, Hvidovre, Denmark;; ^3^Department of Hematology, Roskilde Hospital, Denmark;; ^4^Reference Laboratory for Antimicrobial Resistance and Staphylococci, Statens Serum Institut, Copenhagen, Denmark;; ^5^Department of Clinical Medicine, Faculty of Health and Medical Sciences, University of Copenhagen, Denmark

**Keywords:** bacteremia, *lukF/S-PV*, mortality, Panton-Valentine leucocidin, *Staphylococcus aureus*.

## Abstract

**Background.:**

Panton-Valentine leucocidin is a *Staphylococcus aureus* virulence factor encoded by *lukF-PV* and *lukS-PV* that is infrequent in *S aureus* bacteremia (SAB), and, therefore, little is known about risk factors and outcome of *lukF-PV*/*lukS-PV*-positive SAB.

**Methods.:**

This report is a register-based nationwide observational cohort study. *lukF-PV* was detected by polymerase chain reaction. Factors associated with the presence of *lukF-PV* were assessed by logistic regression analysis. Adjusted 30-day hazard ratios of mortality associated with *lukF-PV* status were computed by Cox proportional hazards regression analysis.

**Results.:**

Of 9490 SAB cases, 129 were *lukF-PV*-positive (1.4%), representing 14 different clonal complexes. *lukF-PV* was associated with younger age, absence of comorbidity, and methicillin-resistant *S aureus*. In unadjusted analysis, mortality associated with *lukF-PV*-positive SAB was comparable to SAB. However, *lukF-PV*-positive SAB nonsurvivors were significantly older and had more comorbidity. Consequently, by adjusted analysis, the risk of 30-day mortality was increased by 70% for *lukF-PV*-positive SAB compared with SAB (hazard ratio, 1.70; 95% confidence interval, 1.20–2.42; *P* = .003).

**Conclusions.:**

*lukF-PV*-positive SAB is rare in Denmark but associated with a significantly increased risk of mortality. Although the risk of *lukF-PV*-positive SAB was highest in the younger age groups, >80% of deaths associated with *lukF-PV*-positive SAB occurred in individuals older than 55 years.

*Staphylococcus aureus* is the most frequent cause of bacteremia worldwide and associated with significant morbidity and mortality [1]. The annual incidence of *S aureus* bacteremia (SAB) in Denmark is approximately 23 per 100 000, and the all-cause 30-day mortality rate has stabilized at 20%–30% [2]. It is well established that SAB short-term mortality is associated with advancing age, comorbidity, as well as type and primary focus of infection. However, risk of death from SAB is multifactorial and dependent on host factors, pathogen-host interactions, and pathogen-specific factors including *S aureus* toxin production [3].

Over the past decades, the Panton-Valentine leucocidin (PVL) toxin has increasingly been associated with *S aureus* infections, and the PVL toxin has been suggested to represent an epidemiological marker of infection severity and outcome in invasive disease [4]. Panton-Valentine leucocidin is an exotoxin belonging to a family of β-barrel-forming proteins. It is composed of two parts, Luk*S*-PV and Luk*F*-PV, which are encoded by the prophage genes *lukS* and *lukF* collective designated *lukF/S-PV*. Panton-Valentine leucocidin is produced by all strains of *S* aureus carrying *lukF/S-PV* [5]. The toxin recruits neutrophil leucocytes through production of interleukins and induces lysis of macrophages, monocytes, and neutrophils [4]. Panton-Valentine leucocidin was initially described in the context of methicillin-susceptibile *S aureus* (MSSA) infections, but it has increasingly been associated with meticillin-resistant *S aureus* (MRSA) [6–8]. The toxin is primarily associated with skin and soft tissue infection (SSTI) and to a lesser extent with invasive disease [4]. A large case-control study and numerous case reports have observed increased short-term mortality in relation to *lukF/S-PV*-positive necrotizing pneumonia, and *lukF/S-PV*-positive SSTI more often required surgical debridement [4, 9, 10]. However, studies have been unable to link *lukF/S-PV* to increased mortality or morbidity in relation to other manifestations of invasive *lukF/S-PV*-positive *S aureus* infections and particularly in the context of community-acquired MRSA [11]. Thus, the impact of *lukF/S-PV* in bacteremia remains unknown [4, 12, 13]. The aims of the present study were to determine the prevalence and risks of PVL-SAB and to quantify the risk of 30-day mortality conferred by PVL.

## MATERIAL AND METHODS

### Study Population

Since 1956, The Danish Staphylococcal Study Group has continuously registered episodes of SAB in Denmark in the nationwide Danish Staphylococcal Bacteremia Database (DSBD) [14]. All SAB isolates are referred for typing, antibiotic susceptibility, and, since 2007, the presence of the *PVL* gene. Only the first SAB episode arising in the same individual was included in the present study. The study was approved by the Danish Data Protection Agency (record no. 2014-41-3376).

### Data Sources

Demographic information was obtained from The Danish Civil Registration System (CRS). This information is updated daily and includes vital status, death, emigration, and loss to follow. Every Danish resident is given a personal identifier at birth or immigration by the CRS, which enables tracking of each individual in multiple registries and eliminates multiple registrations [15]. Data on comorbidity was collected from the National Patient Register (NPR). The NPR is updated monthly and contains information on dates of hospital admission and discharge as well as diagnosis according to the *International Classification of Disease* (ICD). The ICD-8 was used until the end of 1993, and ICD-10 has been used since 1994 [16].

Comorbidity prior to SAB was estimated by the Charlson Comorbidity Index (CCI). In brief, the CCI is a validated score system developed to determine comorbidity in longitudinal studies [17, 18]. It includes 17 diagnostic categories and takes into account both the number and severity of the comorbid diseases. We used the NPR to calculate the CCI score for all SAB cases, and all registrations prior to the SAB hospitalization were used. We defined 3 levels of comorbidity; none (patient without underlying disease), intermediate (CCI 1–2), and high (CCI ≥3). As a proxy for hospital-acquired/healthcare-associated SAB, we used in- or outpatient hospital contact 90 days before the sample date.

### Laboratory Data Collection

Identification of MRSA and *lukF-PV* was performed by polymerase chain reaction (PCR) as previously described using *mecA*, spa, and *lukF-PV* primers [19]. The PCR products were visualized, purified, and sequenced. Ridom StaphType (Ridom, Münster, Germany) and BioNumerics (version 4.6; Applied Maths, Sint-Martens-Latem, Belgium) software, together with the multilocus sequence typing database (http://www.MLST.net), were used for analysis and annotation of the sequences generated from the isolates.

### Statistical Analysis

All values are presented as median and interquartile ranges. χ^2^ statistics was used to compare proportions. Follow-up time was calculated from the index date and was censored on the date of death, emigration, loss to follow-up, or the 19th of January 2015, whichever came first. Logistic regression analysis was used to calculate odds ratio (OR) with 95% confidence interval (95% CI) associated with PVL positivity. Unadjusted and adjusted Cox proportional hazards regression were used to calculate hazard ratio (HR) of mortality with 95% CI. Analysis was performed using IBM SPSS Statistics, version 20.

## RESULTS

Between January 2008 and October 2014, 10508 episodes of SAB were referred to DSBD. A total of 1018 were excluded due to an invalid sample date or invalid/temporary personal identifier. Of the remaining 9490 cases, 129 were *lukF-PV*-positive (1.3%). *mecA* was detected in 148 isolates (1.6%). Twenty-two cases tested positive for *lukF-PV* and *mecA*.

### Characteristics of the Study Population

The majority of patients were >55 years (74.5%), male (62.2%), had had a hospital contact <90 days before the SAB episode (74.2%), and had a CCI score >1 (75.4%) (Table 1). Most episodes of SAB (98.4%), as well as most episodes of *lukF-PV*-positive SAB (83%), were MSSA. However, *lukF-PV* was detected in a higher proportion among MRSA isolates than among MSSA isolates (16.9% vs 1.3 %; *P* < .0001). *lukF-PV*-positive SAB was associated with younger age (56 vs 68 years; *P* = .0001) and was therefore more common in the age group <55 years compared with *lukF-PV*-negative SAB. Two thirds of *lukF-PV*-positive SAB were detected within 90 days after hospitalization compared with 74% of the *lukF-PV*-negative SAB episodes (*P* = .04). Panton-Valentine leucocidin positivity was more common in patients with a low CCI score (44% vs 24%; *P* = .0001). Overall, all-cause 30-day mortality for all SAB episodes was 23.4%, and there was no statistically significant difference between *lukF-PV*-positive and *lukF-PV*-negative SAB episodes in the unadjusted model (24.6% vs 23.4%; *P* = .75).

**Table 1.** Characteristics of Patients With and Without *lukF-PV*-Positive *Staphylococcus aureus* Bacteremia

Variable	All (n = 8666)	*LukF-PV* (n = 129)	Non-*LukF-PV* (n = 8537)	*P* Value
*mecA* positive	142 (1.6%)	21 (16.3%)	121 (1.4%)	.0001
Age, years (IQR)	68 (55–79)	55 (37–68)	68 (56–79)	.0001
Age group (years)				
0–15	470 (5.4%)	12 (9.3%)	458 (5.4%)	
16–35	372 (4.3%)	15 (11.6%)	357 (4.2%)	
36–55	1341 (15.5%)	38 (29.5%)	1303 (15.3%)	
56–75	3451 (39.8%)	43 (33.3%)	3408 (39.8%)	
>75	3032 (35.0%)	21 (16.3%)	3011 (35.3%)	.0001
Male	5405 (62.2%)	82 (63.6%)	5323 (62.4%)	.86
Hospital contact <90 days prior to SAB	6443 (74.3%)	85 (65.9%)	6358 (74.5%)	.03
Charlson Comorbidity Index				
Low	2114 (24.4%)	57 (44.2%)	2057 (24.1%)	
Intermediate	3096 (35.7%)	37 (28.7%)	3059 (35.8%)	
High	3456 (39.9%)	35 (27.1%)	3421 (40.1%)	.0001
Crude 30-day mortality (%)	2033 (23.5%)	31 (24.0%)	2002 (23.5%)	.92

Abbreviations: IQR, median and interquartile range; PVL, Panton-Valentine leucodidin; SAB, *Staphylococcus aureus* bacteremia.

*lukF-PV* cases comprised 14 different clonal complexes (CC) (Supplementary Table 1) compared with 27 different CCs for non-*lukF-PV* cases. The distribution of CCs was significantly different between groups (*P* < .001).

### Factors Associated With *lukF-PV*-Positive *Staphylococcus aureus* Bacteremia

By multivariate logistic regression analysis, younger age (OR, 0.85; 95% CI, 0.41–0.92; per 10 year increment) and *mecA* were associated with *lukF-PV*-positive SAB (OR, 15.09; 95% CI, 9.12–24.97). High comorbidity (CCI ≥3) compared with no comorbidity was associated with a lower odds of *lukF-PV*-positive SAB (OR, 0.60; 95% CI, 0.37–0.97). Hospital contact within 90 days was not associated with PVL-SAB (OR, 0.69; 95% CI, 0.47–1.01) (Table 2).

**Table 2.** Factors Associated With *lukF-PV*-Positive *Staphylococcus aureus* Bacteremia

Variable	Adjusted Odds Ratio (95% CI)	*P* Value
Age, per 10-year increment	0.85 (0.79–0.92)	<.0001
Sex		
Male	1.0	
Female	1.00 (0.69–1.45)	.99
Hospital contact <90 days prior to SAB		
No	1.0	
Yes	0.67 (0.46–0.99)	.04
Charlson Comorbidity Index		
Low	1.0	
Intermediate	0.63 (0.40–1.00)	.05
High	0.59 (0.36–0.97)	.04
*mecA*		
No	1.0	
Yes	13.55 (8.11–22.66)	<.0001

Abbreviations: CI, confidence interval; OR, odds ratio; SAB, *Staphylococcus aureus* bacteremia.

### Factors Associated With 30-Day Mortality After *Staphylococcus aureus* Bacteremia

*lukF-PV*-positive SAB nonsurvivors were older, had more comorbidity, and had had recent hospital contact compared with *lukF-PV*-positive SAB survivors (Table 3). By multivariate analysis adjusting for known risk factors of mortality including age and comorbidity, the adjusted HR of 30-day mortality was 70% higher for *lukF-PV*-positive SAB cases (HR, 1.70; 95% CI, 1.20–2.42) compared with SAB cases without *lukF-PV* (Table 4). A 10-year increment in age increased the risk of mortality by 48% (HR, 1.48; 95% CI, 1.43–1.52), and high and medium comorbidity increased the risk of mortality by 45% and 23% compared with no comorbidity (HR = 1.45, 95% CI = 1.28–1.64 and HR = 1.23, 95% CI = 1.08–1.40, respectively). Recent hospital contact and *mecA* was not associated with 30-day mortality.

**Table 3.** Differences Between 30-Day Survivors and Nonsurvivors With *lukF-PV*-Positive *Staphylococcus aureus* Bacteremia

Variable	All (n = 129)	Non-survivors (n = 31)	Survivors (n = 98)	*P* Value
Age, years (IQR)	55 (37–68)	74 (61–85)	49 (36–63)	.0001
Age group, years				
0–15	12 (9.3%)	1 (3.2%)	11 (11.2%)	
16–35	15 (11.6%)	2 (6.5%)	13 (13.3%)	
36–55	38 (29.5%)	3 (9.7%)	35 (35.7%)	
56–75	43 (33.3%)	10 (32.3%)	33 (33.7%)	
>75	21 (16.3%)	15 (48.4%)	6 (6.1%)	< .0001
Male	82 (63.6%)	16 (51.6%)	66 (67.3%)	.14
Hospital contact <90 days prior to SAB	85 (65.9%)	24 (77.4%)	61 (62.2%)	.13
Charlson Comorbidity Index				
Low	57 (44.2%)	8 (25.8%)	49 (50.0%)	
Intermediate	37 (28.7%)	12 (38.7%)	25 (25.5%)	
High	35 (27.1%)	11 (35.5%)	24 (24.5%)	.06
*mecA*	21 (16.3%)	3 (9.7%)	18 (18.4%)	.40

Abbreviations: IQR, median and interquartile range; SAB, *Staphylococcus aureus* bacteremia.

**Table 4.** Factors Associated With 30-Day Mortality After *Staphylococcus aureus* Bacteremia

Variable	Hazard Ratio (95% CI)	Adjusted Hazard Ratio (95% CI)	*P* Value
*lukF-PV*			
No	1.0	1.0	
Yes	1.08 (0.76–1.53)	1.66 (1.16–2.38)	.006
Age, per 10 year increment	1.47 (1.43–1.51)	1.46 (1.42–1.51)	<.0001
Hospital contact <90 days prior to SAB			
No	1.0	1.0	
Yes	1.09 (0.99–1.20)	1.09 (0.98–1.21)	.11
Charlson Comorbidity Index			
Low	1.0	1.0	
Intermediate	1.74 (1.53–1.96)	1.25 (1.10–1.43)	
High	2.10 (1.86–2.36)	1.49 (1.30–1.70)	< .0001
*mec*A			
No	1.0	1.0	
Yes	1.12 (0.81–1.53)	1.11 (0.80–1.56)	.52

Abbreviations: CI, confidence interval; SAB, *Staphylococcus aureus* bacteremia.

Because pneumonia has been associated with a particularly unfavorable outcome, we performed a subanalysis of the 1982 individuals for whom information on pneumonia was available for the years 2008 and 2009. Of these, 84 (4.7%) individuals had pneumonia and SAB. The increased risk of 30-day mortality associated with *lukF-PV*-positivity was similar in the subset compared with the entire population (HR, 1.98; 95% CI, 1.04–3.78). After adjustment for pneumonia, the risk of 30-day mortality increased for *lukF-PV*-positive individuals (HR, 2.76; 95% CI, 1.26–6.05); *P* = .011) compared with the analysis without pneumonia adjustment. Pneumonia per se was associated with increased 30-day mortality (HR = 1.87, 95% CI = 1.18–2.97, *P* = .008, after adjustment for all other factors included in the primary analysis).

## DISCUSSION

*lukF-PV* was detected infrequently but was associated with a 70% increased risk of 30-day mortality. Deaths associated with *lukF-PV*-positive SAB occurred almost exclusively in individuals older than 55 years of age, although the overall risk of *lukF-PV*-positive *SAB* was highest in the younger age groups. In contrast to other reports, the majority of all episodes of SAB (98%) in our cohort, as well as episodes of *lukF-PV*-positive SAB (83%), were MSSA.

An association between morbidity or mortality in relation to invasive *lukF-PV*-positive *S aureus* infections has not been previously documented with the exception of SSTIs and necrotizing pneumonia [1, 4]. *lukF-PV*-positive SSTIs more frequently required surgical treatment compared with *lukF-PV*-negative infections, and numerous case reports as well as a large prospective case-control study by Gillet and colleagues [4, 9] has showed considerable mortality in relation to PVL-positive pneumonia. However, the study by Gillet and colleagues [4, 9] lacked a comparator group, and more evidence is therefore needed to confirm the findings. Other studies have reported lower illness severity at presentation and shorter hospital stays due to *lukF-PV*-positive disease in general. These findings may be explained by the fact that the sources of *lukF-PV*-positive disease predominantly are SSTIs, which most often are less severe than other manifestations of *S aureus* infection, and that *lukF-PV* is more prevalent among young and healthy individuals [1, 4, 9, 12, 20, 21].

Only a few studies have examined the relationship between *lukF-PV*-positive *S aureus* bacteremia and mortality. In a rabbit model of SAB, short-term mortality was unaffected by PVL [22, 23]. An Australian case-control study conducted by Wehrhahn et al [12] showed a trend towards an increased 30-day mortality among *lukF-PV*-positive SAB cases compared with PVL-negative SAB cases (16% vs 10%). Seybold et al [13] showed a lower crude in-hospital mortality among *lukF-PV*-negative SAB (8%) compared with *lukF-PV*-positive SAB (29%). However, Chen and colleagues [24, 25] failed to show an association between *lukF-PV*-positive SAB and mortality.

The discrepancy between our findings and other studies may be explained by the relatively low number of *lukF-PV*-positive cases in previous studies and lack of confounder adjustment in some studies. Furthermore, most studies included only cases with MRSA that per se may be associated with an increased risk of death.

Overall, *lukF-PV*-positive *S aureus* in blood cultures varies between 0% and 40% and is mainly described in the context of MRSA and community-acquired disease [3, 13, 26–28]. Reports from Northern Europe and the United Kingdom confirm the low prevalence of *lukF-PV* (0%–2.1% of SAB isolates) observed in our study [29–31]. Based on these numbers, PVL toxin testing should not be routinely recommended but may be considered among the elderly above 55 years of age and in younger patients presenting with symptoms compatible with necrotizing pneumonia [9].

The present study has several strengths, including the large size of the study population, longitudinal and population-based design, and the uniform registration system. In addition, the high prevalence of MSSA compared with MRSA may have eliminated some possible confounding factors associated with MRSA infection. Limitations of the study include lack of information on infection severity, source, presence of polymicrobial infection, and antimicrobial treatment strategy. In addition, possible errors in the discharge data upon which the comorbidity scores were constructed may also confound the results. However, the misclassification bias is thought to be nondifferential between *lukF-PV*-positive and *lukF-PV*-negative SAB episodes. Estimation of comorbidity by the CCI also has several limitations such as failure to include all comorbid diseases and sufficiently grade the severity of illness. Furthermore, hospital contact within 90 days before the SAB episode was used as a proxy for hospital/healthcare-acquisitioning, which may limit comparison with other studies. Finally, the low number of PVL SAB may have limited the statistical power of the analysis.

## CONCLUSIONS

In the present study, we demonstrate that *lukF-PV*-positive SAB is rare in Denmark, but it is associated with a considerable increased risk of 30-day mortality when adjusting for well known risk factors of SAB-related mortality. The prevalence of *lukF-PV*-positive SAB was most abundant among the younger age groups; however, the majority of deaths associated with *lukF-PV*-positive SAB occurred among individuals above 55 years of age. Panton-Valentine leucocidin is still subject to much debate, and more research is needed to understand the role of PVL in invasive diseases such as bacteremia.

## Supplementary Data

Supplementary material is available at *Open Forum Infectious Diseases* online.

Supplementary Data
