# Supplementary material for: Increased Age-Dependent Risk of Death Associated With lukF-PV-Positive Staphylococcus aureus Bacteremia
Source: Open Forum Infect Dis. 2016 Dec 5;3(4):ofw220. doi: 10.1093/ofid/ofw220 (PMC5146761; doi:10.1093/ofid/ofw220)
Supplement: Supplementary Data [file supp_3_4_ofw220__index.html]

Increased Age-Dependent Risk of Death Associated With lukF-PV-Positive Staphylococcus aureus Bacteremia — Supplementary Data 

# Increased Age-Dependent Risk of Death Associated With *lukF-PV*-Positive *Staphylococcus aureus* Bacteremia

## Supplementary Data

Data files

- Supplementary Data - Supplementary Data
