# Supplementary material for: Increased Age-Dependent Risk of Death Associated With lukF-PV-Positive Staphylococcus aureus Bacteremia
Source: Open Forum Infect Dis. 2016 Dec 5;3(4):ofw220. doi: 10.1093/ofid/ofw220 (PMC5146761; doi:10.1093/ofid/ofw220)
Supplement: Supplementary Data [file supp_ofw220_Supplemental_table_1.docx]

**Supplemental table 1. Clonal complexes of *Staphylococcus aureus* bacteremia with or without *lukF-PV***

|  | ***lukF-PV*** | |
| --- | --- | --- |
| Clonal complex (CC) | Yes  (n = 129) | No  (n = 8537) |
| CC30 | 33 (25.6%) | 1231 (14.4%) |
| CC1 | 12 (9.3%) | 630 (7.4%) |
| CC121 | 9 (7.0%) | 71 (0.8%) |
| CC15 | 8 (6.2%) | 951 (11.1%) |
| CC8 | 7 (5.4%) | 574 (6.7%) |
| CC22 | 7 (5.4%) | 274 (3.2%) |
| CC80 | 7 (5.4%) | 9 (0.1%) |
| CC45 | 5 (3.9%) | 1729 (20.3%) |
| CC88 | 5 (3.9%) | 28 (0.3%) |
| ST 152/377 | 5 (3.9%) | 0 |
| CC5 | 4 (3.1%) | 623 (7.3%) |
| CC59 | 3 (2.3%) | 188 (2.2%) |
| CC97 | 3 (2.3%) | 161 (1.9%) |
| CC398 | 1 (0.8%) | 77 (0.9%) |
| Other | - | 1041 (12.2%) |
| Unknown | 9 (7.0%) | 374 (4.4%) |
| Not typed | 11 (8.5%) | 576 (6.7%) |
